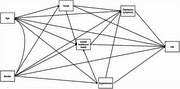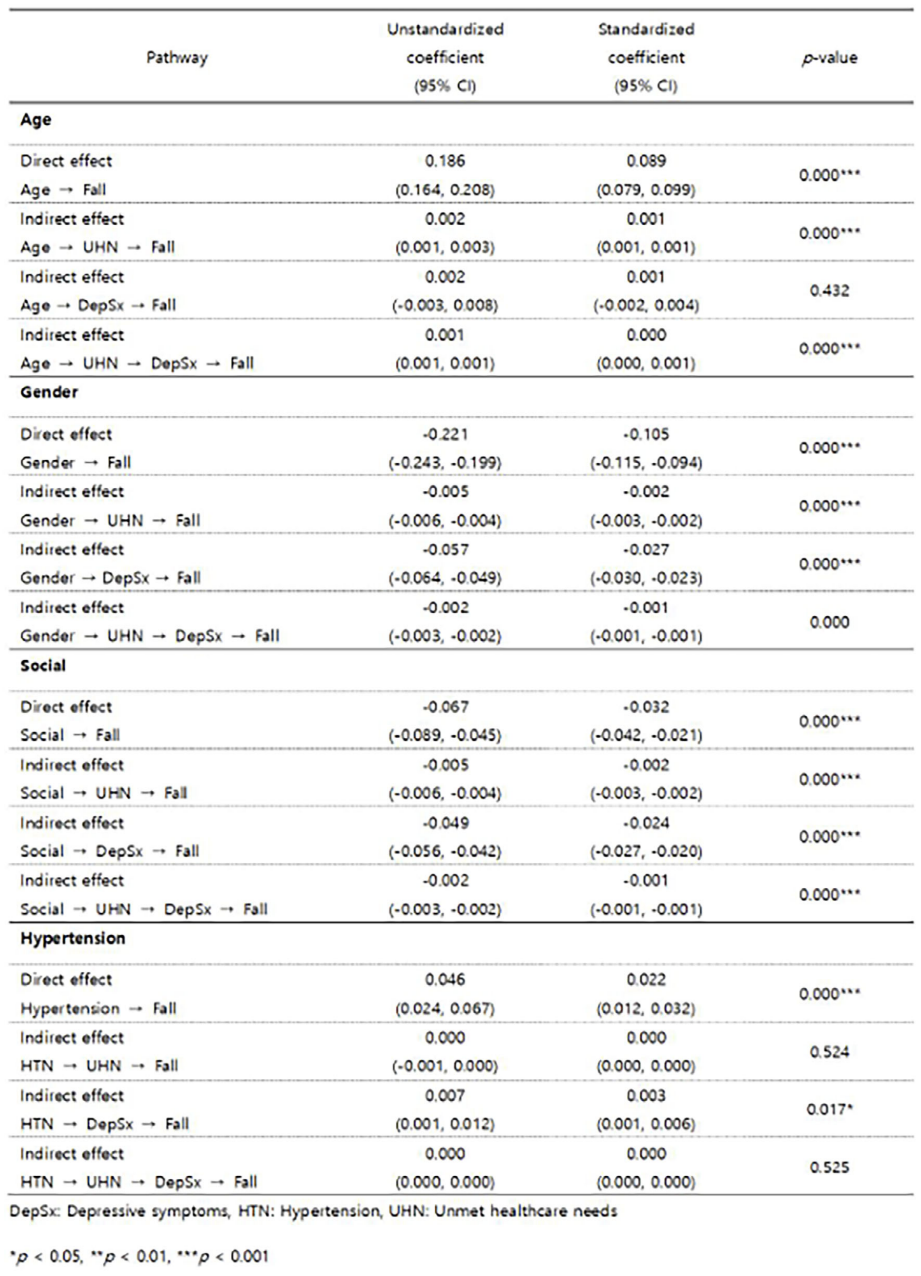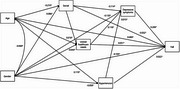# Analysis of Factors Influencing Falls Trough the Mediating Effects of Depressive Symptoms and Unmet Healthcare Needs: Focusing on Demographic Characteristics, Social Activity, and Hypertension

**DOI:** 10.1002/alz70860_099522

**Published:** 2025-12-23

**Authors:** Jiwon Hong, Ickpyo Hong

**Affiliations:** ^1^ Yonsei University Mirae Campus, Wonju‐si, Gangwon‐do, Korea, Republic of (South); ^2^ Yonsei University, Wonju, Gangwon‐do, Korea, Republic of (South)

## Abstract

**Background:**

Falls are a significant issue among older adults, leading to physical injuries and a decline in quality of life. Previous studies suggest that depression symptoms increases the risk of falls, and unmet healthcare needs are reported as factors that can cause diseases or exacerbate chronic conditions, potentially leading to physical harm. Therefore, this study aims to analyze variables related to falls and to examine the mediating effects of unmet healthcare needs and depression symptoms in relation to falls.

**Method:**

This study utilized data from the 2023 Community Health Survey in South Korea and analyzed 81,794 older adults aged 65 years and above. The exposure variables were factors related to falls, including age, gender, social activities, and hypertension, while the mediating variables were depression symptoms and unmet healthcare needs. Path analysis was conducted with falls set as the outcome variable.

**Result:**

Among the participants, 43,957 individuals (53.74%) were aged 65–74, while 37,837 individuals (46.26%) were aged 75 or older. Males accounted for 34,882 individuals (42.65%), while females were more prevalent at 57.35%. The model fit was determined to be adequate (chi‐square value = 15.565; RMSEA = 0.013; CFI = 0.999; TLI = 0.975; SRMR = 0.002). Age, gender, and social activities showed indirect associations with falls through unmet healthcare needs; however, hypertension showed no statistically significant association, as the coefficient value was close to 0 (hypertension→unmet healthcare needs→falls: coefficient = 0.000, *p*‐value = 0.524). On the other hand, gender, social activities, and hypertension all demonstrated indirect associations with falls through depression symptoms, whereas age did not show an indirect association with falls through depression symptoms (age→depression symptoms→falls: coefficient = 0.002, *p*‐value = 0.432).

**Conclusion:**

Unmet healthcare needs were found to mediate the relationship between age, gender, social activities, and falls, while depression symptoms mediated the relationship between gender, social activities, hypertension, and falls. These findings suggest the need for interventions to alleviate depression symptoms and improve medical accessibility to prevent falls among older adults.